# Xylem structure and hydraulic characteristics of deep roots, shallow roots and branches of walnut under seasonal drought

**DOI:** 10.1186/s12870-022-03815-2

**Published:** 2022-09-14

**Authors:** Lin Wang, Yongxin Dai, Jinsong Zhang, Ping Meng, Xianchong Wan

**Affiliations:** 1 College of Forestry, Shanxi Agricultural University, Taigu, Shanxi 030801 People’s Republic of China; 2 Institute of New Forestry Technology, Chinese Academy of Forestry, Beijing, 100091 People’s Republic of China; 3 Research Institute of Forestry, Chinese Academy of Forestry, Beijing, 100091 People’s Republic of China

**Keywords:** Xylem anatomy, Vulnerability to cavitation, Deep and shallow roots, Mechanism of embolism repair, Xylem conduits, Hydraulic conductivity, Dry and wet season

## Abstract

**Background:**

Despite the importance of root hydraulics, there is little research on the in situ dynamic responses of embolism formation and embolism repair of roots distributed in different soil depths in response to different water regimes.

**Results:**

The vessel diameter, hydraulic conductivity, and vulnerability to cavitation were in the order of deep root > shallow root > branch. The midday PLC of shallow root was the highest in the dry season, while the midday PLC of deep root slightly higher than that of branch with no significant difference in the two seasons. The capacity of embolism repair of roots was significantly greater than that of branch both in dry season and wet season. The xylem pressure was in the order of deep roots > shallow root > branch, and it was negative in most of the time for the latter two in the dry season, but positive for both of the roots during the observation period in the wet season. The NSC and starch content in roots were significantly higher than those in branches, especially in the dry season. In contrast, roots had lower content of soluble sugar.

**Conclusions:**

The relatively stable water condition in soil, especially in the deep layers, is favorable for the development of larger-diameter vessels in root xylem, however it cannot prevent the root from forming embolism. The mechanism of embolism repair may be different in different parts of plants. Deep roots mainly depend on root pressure to refill the embolized vessels, while branches mainly depend on starch hydrolysis to soluble sugars to do the work, with shallow roots shifted between the two mechanisms in different moisture regimes. There is theoretically an obvious trade-off between conducting efficiency and safety over deep roots, shallow roots and branches. But in natural conditions, roots do not necessarily suffer more severe embolism than branches, maybe due to their root pressure-driven embolism repair and relatively good water conditions.

**Supplementary Information:**

The online version contains supplementary material available at 10.1186/s12870-022-03815-2.

## Background

The function of plant stems and roots can affect the growth and survival of plants, among which water absorption in roots and water transport in roots and stems are the most important to ensure the survival of plants [1]. Many studies have formalized the water transport characteristics of plants from base to top, but the roots are relatively lagging behind. Previous studies have proved that deep roots are important to plants in adversity such as drought, and in some ecosystems, deep roots can contribute more than 75% of transpiration water consumption during the dry season [2]. However, the physiological mechanism of maintenance of deep roots’ water transport is still unclear, which limits our thorough understanding of the hydraulic structure of trees.

The morphology of water transport tissue is closely related to the ability and safety of water transport. It is well known that both the capacity of xylem to transport water (hydraulic efficiency) and to resist embolism (hydraulic safety, opposite to vulnerability to embolism (gas emboli enters a conduit under more negative pressure, and expands and fills the conduit lumen) is potentially related to conduit diameter [3]. There exists a phenomenon that within individuals the conduit diameter widens from the stem tip towards the base even into the roots ("basipetal widening"), which has been found in numerous species and regarded as a pervasive and adaptive pattern [4–6]. Natural selection should favor minimizing the hydraulic resistance that increases with stem growing longer and the resultant elongation of water conductive path, thus the essential driver of this variation pattern is plant height, fitted well to a power function, followed by climate [7–9]. Based on Hagen-Poiseuille's law, the increase or decrease of vessel diameter has a great influence on the hydraulic conductance or resistance. Therefore, roots are conferred to wider conduits and hence higher hydraulic conductivity. The relationship between efficiency and diameter is well understood and widely accepted [3]. It is undoubted that there is a trade-off between vulnerability to freezing-induced embolism and conduit diameter, but as for drought-induced embolism, a vulnerability-diameter link is ambiguous and lack of robust theory and evidence support [10]. If such a vulnerability-diameter link exists, there would be an efficiency-safety tradeoff gradient along plant height. So far, evidences about the hydraulic characteristics of different organs within a plant are inconsistent, especially for roots distributed in different depths. Some researchers have reported that vessel diameter of deep roots is not different from, even smaller than that of shallow roots in some species [11, 12]. Coarse roots are generally thought to be more vulnerable to embolism, but there is opposite evidence. In addition, roots are usually in a more abundant and stable (not dramatic variation of soil water content) water environment than shoots, while the diameter and length of root vessels are usually greater than those of shoot vessels. Thus, it is interesting to know whether roots of different depths are more vulnerable to embolism than shoots. Unfortunately, there is little research on the issue [13].

At present, the physiological mechanism of embolism repair is not fully resolved, and actually is under heated debate [14, 15]. Embolism repair mainly includes seasonal repair and daily repair. Seasonal repair is a long process, which occurs in trees confronted with freezing events and is generally believed to rely mainly on root pressure and the regrowth of new conductive tissues [16]. And for the latter it is currently recognized that embolism repair mainly includes root pressure-driven embolism repair and osmoregulation mediated active repair under tension ('novel refilling') [17–19]. There is evidence that novel refilling also occurs during seasonal repair. For root pressure, recent studies have verified that only a few dozen kilopascals can be maintained, and it is far from enough to fully repair embolism of tall trees [20–22]. Nevertheless, root pressure may play an important role for embolism repair in the dwarf plants or low part of trees. In a previous study, it was interestingly found that the embolism repair in poplar basal part of stem after drought is mainly related to root pressure [23], thus, whether root pressure plays an important role in root embolism repair still needs more experimental evidences. For the tall part of trees, it is necessary to rely on the osmotic mechanism to repair the embolism. Osmoregulation mediated embolism repair is generally believed to be accompanied by the release of osmoregulatory substances from parenchyma cells around the vessel into the embolized vessel [16, 24]. The osmotic pressure gradient drives water into the embolized vessel and completes the embolism repair. Osmoregulatory repair is usually accompanied by the conversion of starch to sugar and the transport of sugar into the embolized vessel, and this phenomenon has only been recently reported [25]. Also, aquaporin plays an important role in the process of embolism repair [26]. Therefore, the exact mechanism of embolism repair in different parts of a tree needs further experimental evidence.

Nonstructural carbohydrates (NSC) consist of soluble sugar and starch, and play an important role as a buffer in maintaining carbon balance between carbon uptake and utilization [27, 28]. NSC also plays an important role in plant water transportation, such as maintaining osmotic pressure, participating in embolism repair [29], promoting root growth and increasing root absorbing ability. The roots are the farthest away from the photosynthetic organs of plants, especially the deep roots. Thus, roots may be vulnerable to carbon deficit, for example, drought can limit phloem transport function, resulting in carbon starvation in roots [30, 31]. It is not clear whether there are differences in carbon metabolism between deep roots and shallow roots under the condition of photosynthesis limited by drought, and whether the differences in carbon metabolism affect the water absorption and transportation function of roots.

Walnut is an important economic tree species widely planted in north and northwest of China, and has strong environmental adaptability and drought resistance [32]. Further understanding of water utilization and carbon metabolism of walnut is helpful for efficient cultivation of walnut. In this study, walnuts growing in the southern foothill of Taihang mountains were taken as the research materials. In this region, there are obvious dry and wet seasons in a year under the influence of the Asian monsoon. By comparing the differences in morphology, water transport capacity and embolism vulnerability between deep and shallow roots of walnut, the change dynamics of embolism repair ability and NSC content of deep and shallow roots under seasonal drought conditions were analyzed. We focused on the following questions: (1) Is there any trade-off between conducting efficiency and safety over deep roots, shallow roots and branches? (2) Are root water permeability, embolism vulnerability and root vessel anatomy in different soil layers correlated? (3) What are the changes of embolism formation, embolism repair ability, and embolism repair mechanism of roots distributed in different soil depths in dry and wet seasons? (4) Whether the NSC is involved in the embolism repair?

## Results

**Fig. 1:**
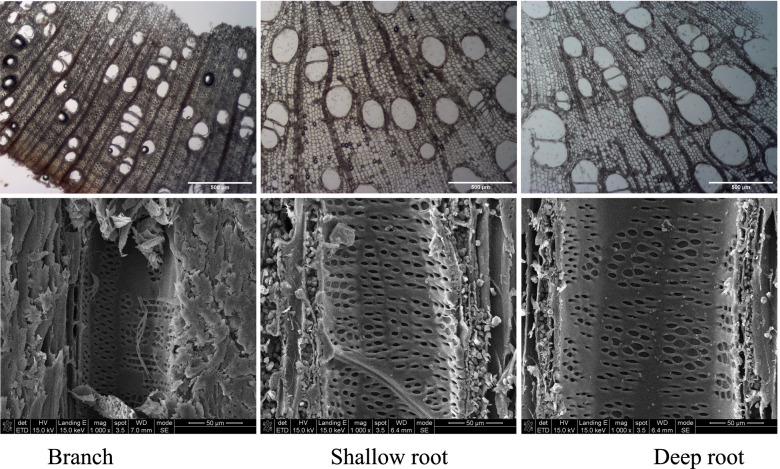
Xylem cross sections of branches, shallow roots, and deep roots under optical microscope (upper row), and vessel wall micrographs of branches, shallow roots, and deep roots with SEM (lower row). Three biological replicates for each plant organ were sectioned, and two visual fields of each replicate were chosen to be photographed for analysis

The mean vessel diameter in deep and shallow roots was 153.01 ± 41.12 and 124.83 ± 32.19 μm respectively, and significantly larger than that in branches, 64.71 ± 19.02 μm (P < 0.05). The mean vessel diameter in deep roots was slightly wider than that in shallow roots, but the difference was not significant (Table 1, Fig. 1). Vessel density of branches was significantly greater than that of both deep and shallow roots, and the vessel density of deep root was slightly higher than that of the shallow root, but the difference was not significant. There were significant differences in the vessel area ratio among the three different materials (*P* < 0.05), and the ratio was in the order of deep root > shallow root > branches (Table 1).

**Table 1:** Vessel diameter, vessel density, pit area and pit area ratio of walnut branches, shallow roots, and deep roots

	Branch	Shallow root	Deep root
Mean vessel diameter (um)	64.71 ± 19.02 b	124.83 ± 32.19 a	153.01 ± 41.12 a
Vessel density (N mm^−2^)	60.75 ± 4.85 a	34.67 ± 3.51 b	36.67 ± 4.16 b
Vessel area ratio %	13.1 ± 0.56 c	18.2 ± 2.42 b	24.6 ± 2.49 a
Mean pit pore area (μm^2^)	2.88 ± 0.85 c	7.54 ± 2.38 b	16.32 ± 8.6 a
Max pit area (μm^2^)	4.25	12.69	30.49
Min pit area (μm^2^)	1.56	5.09	7.54
Pit area ratio (%)	14.3 ± 2.3 b	16.7 ± 2.6 b	29.1 ± 4.7 a

Means ± standard deviations (*n* = 3) are shown, and different letters refer to significant difference at *P* < 0.05

**Fig. 2:**
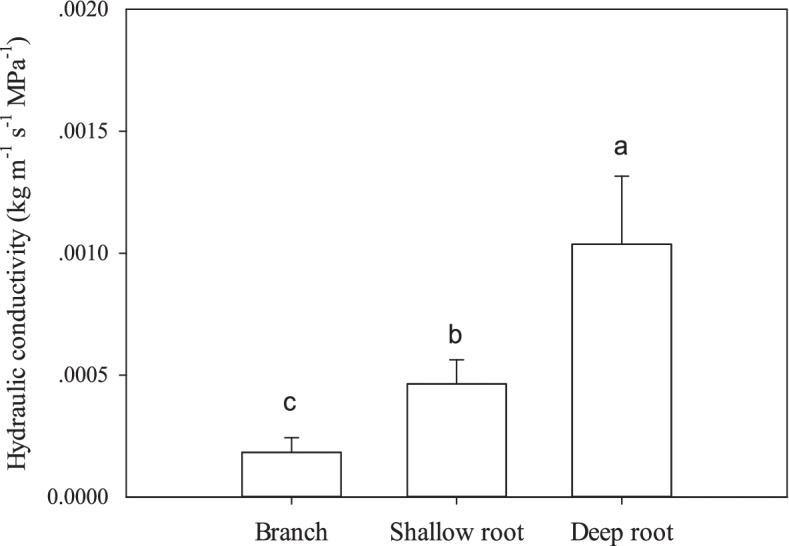
Axial guide hydraulic conductivity of walnut branches, shallow roots and deep roots. Means ± standard deviations (*n* = 3) are shown, and different letters refer to significant difference at *P* < 0.05

**Fig. 3:**
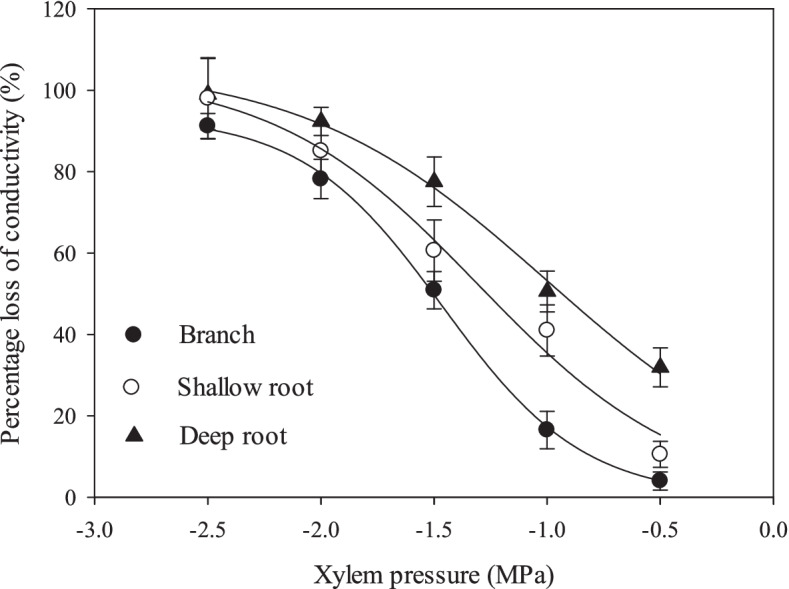
The vulnerability curves of branches, shallow roots and deep roots. Means ± standard deviations (*n* = 3) are shown

The axial hydraulic conductivity of shallow roots, deep roots and branches was 4.64 ± 0.99 × 10^–4^, 10.37 ± 2.80 × 10^–4^, and 1.83 ± 0.60 × 10^–4^ kg m^−1^ s^−1^ MPa^−1^, respectively, showing deep roots had the significantly higher hydraulic conductivity than shallow roots and branches (Fig. 2). Embolism vulnerability order was as the same as hydraulic conductivity: deep root > shallow root > branch (Fig. 3). The P_50_ (water potential at 50% loss in hydraulic conductivity) of branch, shallow root and deep root was -1.556 ± 0.035, -1.276 ± 0.063, and -0.966 ± 0.056 MPa, respectively. There was a trade-off between conductivity efficiency and drought resistance.

**Fig. 4:**
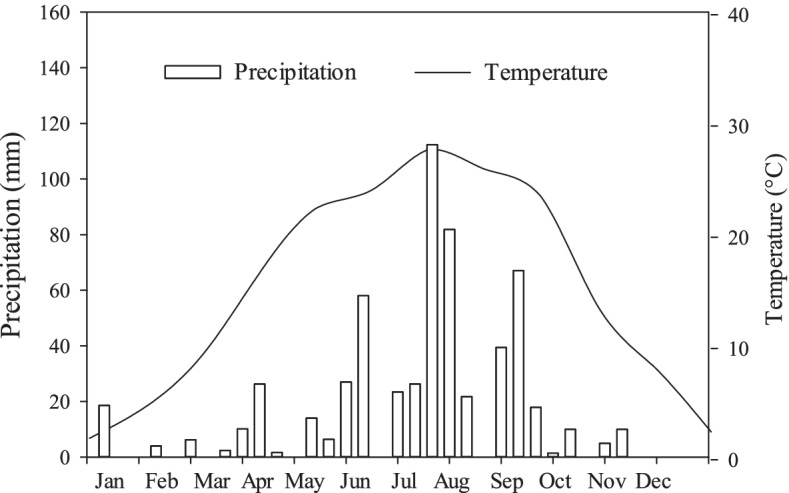
Temperature and precipitation of the test site in 2017

In 2017 when this experiment was conducted, the precipitation in this region was 205 mm from January 1^st^ to mid-July, and 436 mm from mid-July to the end of December (Fig. 4). In the dry season, the moisture content in the shallow soil layers was significantly lower than that in the deep soil (*P* < 0.05), however in the wet season there was no significant difference in moisture content between the shallow and deep soil layers (Table 2). The soil water content was greatly affected by the seasonal precipitation, for the soil moisture content in the dry season was only about 60% of that in the wet season, especially in the shallow layers (Fig. 4; Table 2).

**Table 2:** Soil water contents at different depths in dry and wet season in 2017

Soil Depth	Dry season	Wet season
15 cm	12.58 ± 0.49 a	23.38 ± 0.86 a
60 cm	14.23 ± 0.91 b	22.23 ± 0.78 a

Means ± standard deviations (*n* = 3) are shown, and different letters refer to significant difference in the same season at *P* < 0.05

**Fig. 5:**
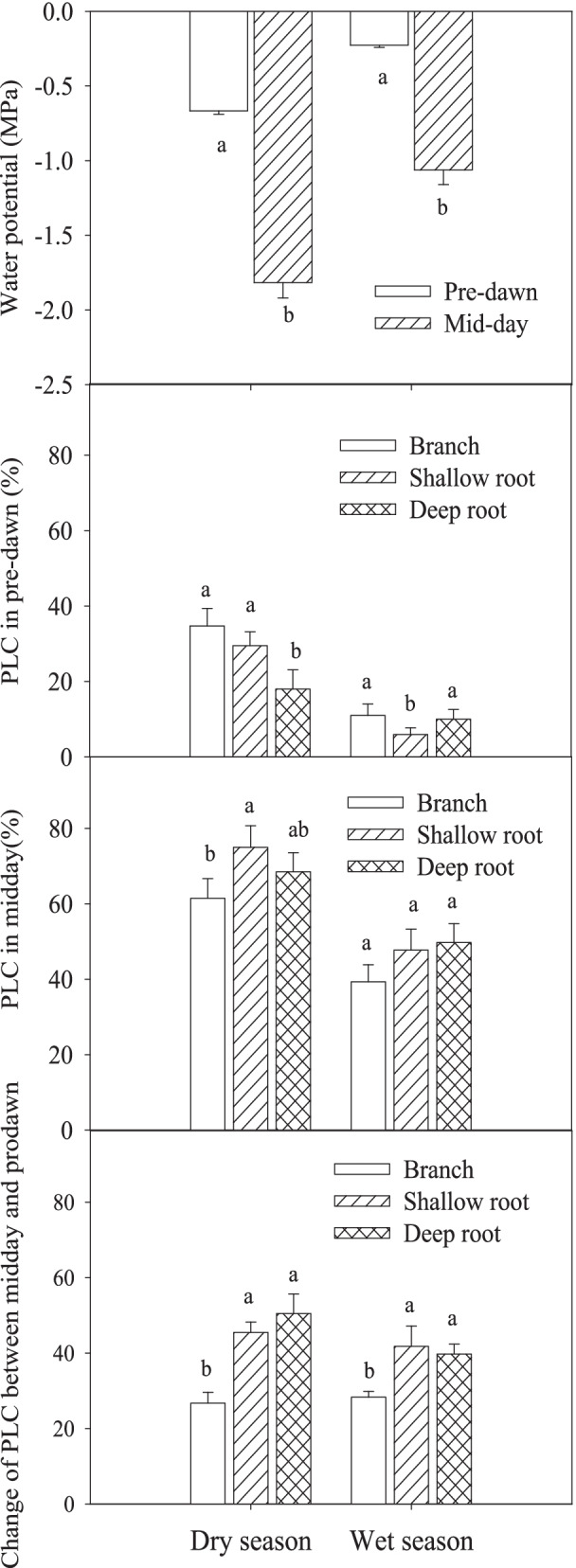
The twig water potential, predawn and midday PLC of branches, shallow roots and deep roots. Means ± standard deviations (*n* = 3) are shown, and different letters refer to significant difference in the same season at *P* < 0.05

Both predawn and midday water potential of twigs was significantly lower in dry season than that in wet season (*P* < 0.05), and midday water potential was lower than predawn one in both seasons (Fig. 5). The midday water potential in the dry season and wet season were -1.91 MPa and -1.06 MPa, respectively, while the pre-dawn water potential was -0.77 and -0.22 MPa in the dry season and wet season. In dry season, the predawn PLC of branches and shallow roots was significantly higher than that of deep roots (*P* < 0.05), but the midday PLC of shallow roots was the largest and significantly higher than that of branches (*P* < 0.05) (Fig. 5). The recuperation of branch PLC between noon and predawn was significantly lower than that of the shallow root and the deep root (*P* < 0.05), and the recuperation degree of PLC in deep roots was slightly higher than that in shallow roots, but the difference was not significant. In wet season, the predawn PLC of shallow roots was significantly lower than that of deep roots and branches, and there was no significant difference in predawn PLC between deep roots and branches. The midday PLC of branches was slightly lower than the other two, but there was no significant difference in midday PLC among the three organs. The results showed that the diurnal variation of root PLC was significantly greater than that of branches in both dry and wet seasons.

**Fig. 6:**
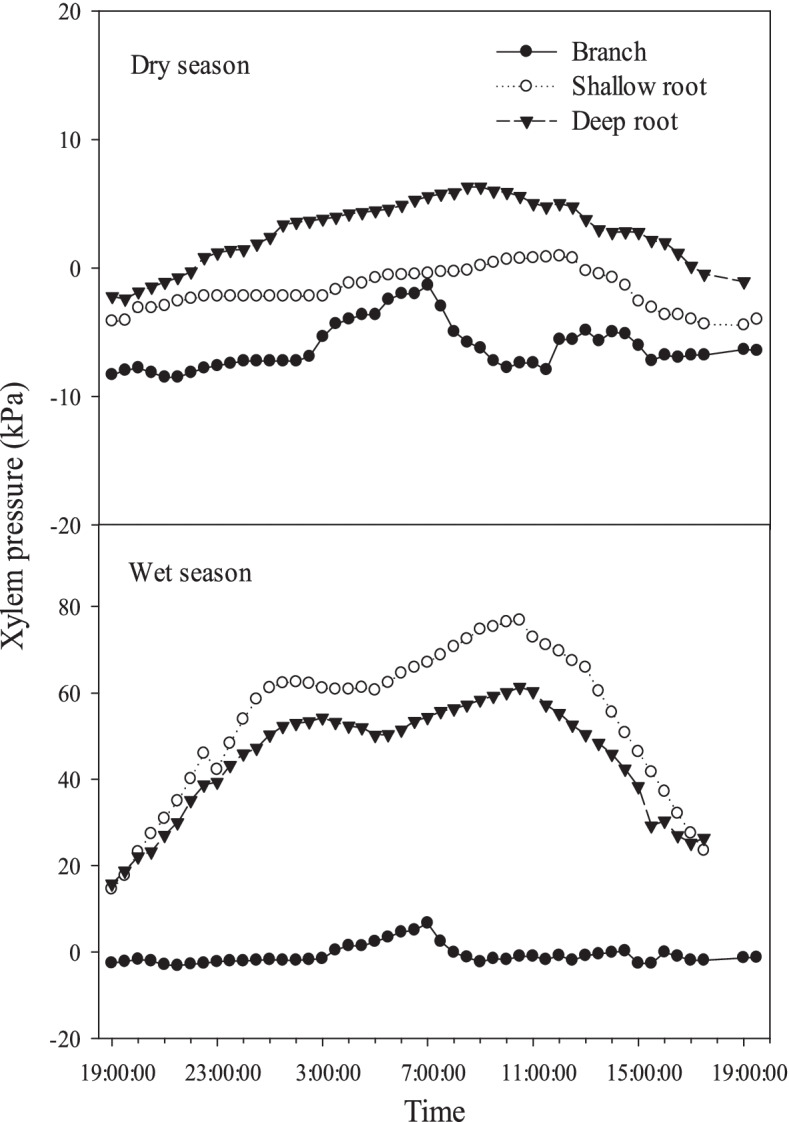
The xylem pressure of branch, shallow root and deep root in dry and wet season. Means ± standard deviations (*n* = 3) are shown

In the dry season, the order of xylem pressure from different organs was deep root > shallow root > branch, but in the wet season, the order changed to shallow root > deep root > branch (Fig. 6). Compared to that in the dry season, the xylem pressure of all the organs in wet season increased to different degrees. The xylem pressure increased more in deep and shallow roots, and less in branches. In the dry season, xylem pressure of deep roots did not exceed to 10 kPa, and the xylem pressure of shallow roots and branches was negative in most of the time. However, in the wet season, the xylem pressure of deep and shallow roots reached to 60–80 kPa, and maintained positive during the observation period, and their maximum values occurred around 11:00 am.

**Fig. 7:**
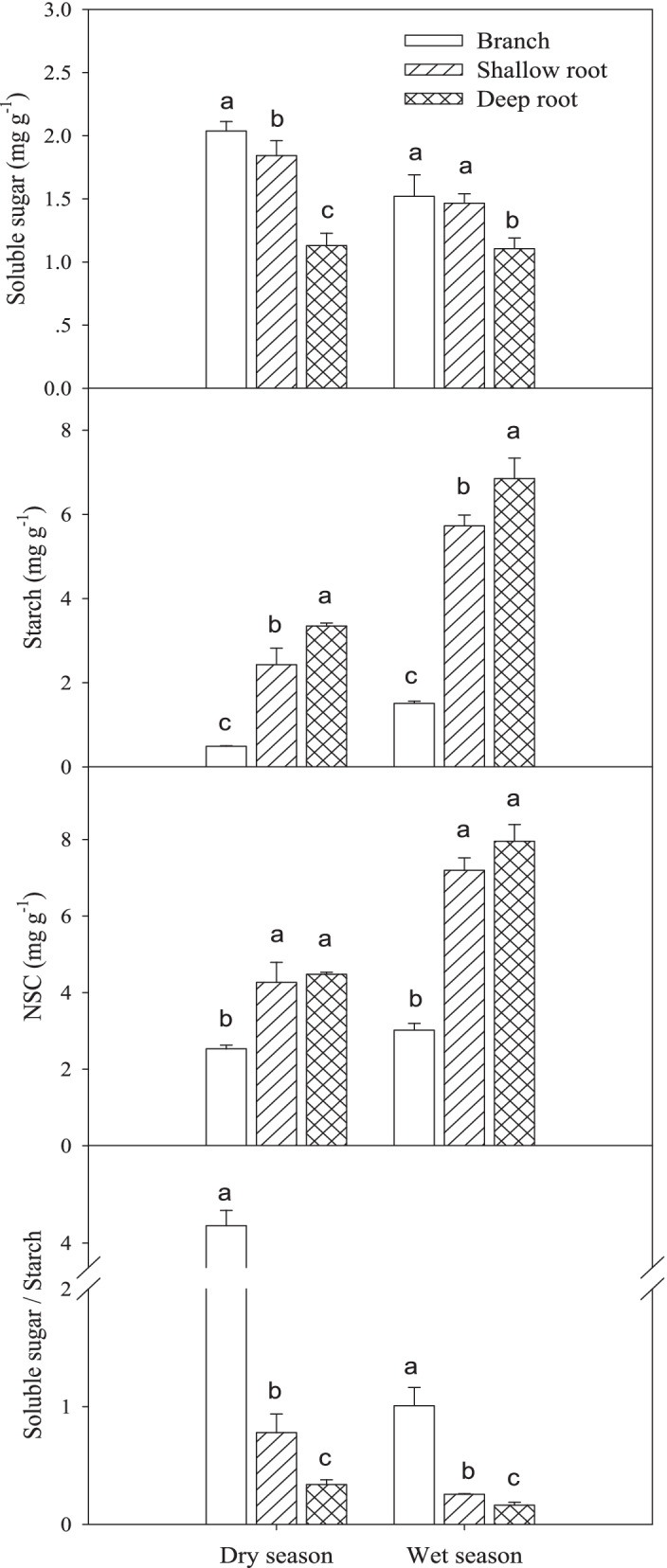
The NSC contents of branch, shallow root and deep root in dry season and wet season. Means ± standard deviations (*n* = 4) are shown, and different letters refer to significant difference in the same season at *P* < 0.05

In both dry and wet season, there were significant differences in soluble sugar contents among the different parts (*P* < 0.05), and the order of the soluble sugar contents was branch > shallow root > deep root (Fig. 7). On the contrary, starch concentration order was deep root > shallow root > branch. In dry season, NSC content of both deep and shallow root was significantly higher than that of branches by 77% and 69%, respectively. The soluble sugar/starch ratio was in the order of branch > shallow root > deep root, and the ratios were 4.14, 0.78, and 0.34 in the dry season, and 1.01, 0.26, and 0.16 in the wet season. The soluble sugar/starch ratio was reduced in wet season. The soluble sugar content in the wet season was lower than that in the dry season, soluble sugar content of branches, shallow roots and deep roots in the wet season was 74%, 79% and 97% of that in the dry season, respectively. The NSC content of roots increased significantly in the wet season, and the NSC content of shallow roots and deep roots was increased to 136% and 208% of that in the dry season, The increase was mainly contributed by starch content.

## Discussion

Our first question was whether root water permeability, embolism vulnerability and root vessel anatomy were correlated in different soil layers. In this study, we compared vascular tissue microanatomical and hydraulic characteristics among the deep roots, shallow roots and branches, and the results showed that the vessel diameter increased from shoot to root, and along with the increase of soil depths, as a result, the deep roots had the greatest axial hydraulic conductivity, followed by shallow roots and then branches. Although we did not measure the distance from the tree top in each sample, the vessel diameters in different parts of plants agreed with the tip-to-base xylem conduit widening pattern [4]. Generally, vessel density is inversely proportional to vessel diameter, but deep roots in this study also had slightly greater vessel density than the shallow roots. The water environment may play an important role for this phenomenon. The water content in deep soil layers was relatively stable, significantly higher than the shallow soil during the dry season, which ensures that deep roots are in a relatively better water condition. By contrast, the water content of shallow soil layers changed sharply, which makes shallow root system suffer from severe drought stress during the dry season, and results in the decrease of shallow root vessel diameter due to its response to drought stress [33]. If the conduit diameter of shallow roots is larger, their water delivery systems may collapse at noon in the dry season. It can be seen that the fluctuation of soil moisture and the strong transpiration demand in summer are the main factors limiting the size of root conduits. In addition, root conduit diameter may be changed by an adaptation strategy due to the winter freeze–thaw cycles [13]. In this study, the region also has permafrost of 20–30 cm thick, thus, the winter freeze–thaw cycles cannot be ruled out as an influence factor. To cope with the winter cold, the shallow root may retard its vessel diameter development to avoid freeze–thaw cavitation in winter. By contrast, the deep root can develop large vessel diameters with small seasonal water fluctuation and without freeze in winter, which is also suitable to the carbon saving strategy because the increase of vessel size requires less carbon investment relative to the increase of vessel density [34]. Unlike the aboveground part, the roots need less mechanical strength, which can reduce mechanical strength investment resulting in more investment to conduits for water transport.

Roots predictably have higher hydraulic conductivity according to the tip-to-base xylem conduit widening pattern, as evidenced in this study. On the other hand, vulnerability curve detection showed that the deep root was the most vulnerable, followed by the shallow root and then the branch, seeming to show an efficiency-safety tradeoff gradient corresponding to conduit diameter widening along the stem. Tip-to-base xylem conduit widening pattern predicts that variations of conduit diameter, conduit length and pits structure must concur to prevent any single component from resulting in disproportionate total conduit hydraulic resistance [7]. Our results also show that pit area ratio of vessels in the most recently formed ring and the maximum pit area are in the order of deep roots > shallow roots > branches. According to the 'rare pit hypothesis', when there are more pits in larger vessels, they are more likely to have a big leaky pit, leading to air-seeding embolism [35]. Efficiency-safety tradeoff has been extensively studied, however, no consensus is reached with tradeoffs being weak or nonexistent [36, 37]. The efficiency-safety tradeoff theoretically exists, because the structural characteristics of the xylem network determine that plants face a xylem level tradeoff to transport as much water as possible while minimizing the risk of drought-induced embolism [38]. But at large scale, e.g. the whole conduits or the plant, this tissue-level tradeoff could be weakened [38]. As plants adapt to the environment, changes in morphological or anatomical structures result in the efficiency or safety correlated to many factors [37].

Moreover, within individual plants, organs may not behave as predicted from the efficiency-safety tradeoff relationship. In this study, either in dry season or wet season, PLC of deep roots did not show significant increase compared to that of the other two organs, indicating no obvious sign of susceptibility to embolism. This is partly because the water potential of deep roots is inevitably higher than that of branches, and partly because the deep roots repair their embolism frequently through root pressure (discussed below). According to the hydraulic limitation hypothesis, water supply to the leaves becomes more difficult as the tree height increases, due to the decreases of water potential from root to leaf caused by the increase of gravity and friction along plant height [39]. Thus, different organs are endowed with different hydraulic functions. The distal organs (leaves or terminal shoots) are easily sacrificed under drought to maintain the hydraulic integrity of stem (known as "hydraulic segmentation" or "hydraulic fuse") [40]. Fine roots also readily detach during drought as their lower construction costs and ability to grow quickly, playing a "hydraulic fuse" role [41]. In that case deep root is indeed more vulnerable to embolism if only embolism resistance is compared. But for whole-plant level, it is an integrated result by coordination of the hydraulic functions of each organ.

Our second question concerned the changes of embolization, embolism repair ability, and the mechanism of embolism repair of roots distributing different soil depths in dry and wet seasons. In this study, the midday PLC of roots did not show severer than that of branches regardless in dry season or in wet season except shallow root in dry season. Although the soil water is relatively stable, especially in the deep soil, the deep roots also have embolism at noon in the summer with strong transpiration. The phenomena in this study once more indicates that the xylem of trees is a continuous system from roots to leaves [20], and the signal of leaf water potential must be transmitted to the root system. On the other hand, the water absorption of roots is mainly restricted by the radial movement of water in roots and in this process, water needs to flow across the membrane ([42], also see supplemental Fig. 1), so that the water absorption of root system usually cannot catch up with the transpiration loss of water in dry season. As a result, also considering the greater vulnerability of roots, the xylem of root system also establishes tension under transpiration, resulting in cavitation in root conduits. As for shallow root in dry season, the significantly lower water content in shallow soil layer and strong transpiration contributes to its highest midday PLC.

The maintenance of xylem hydraulic structure is mainly achieved by two ways. One is cavitation resistance. Generally speaking, tree species with strong cavitation resistance, for which the vulnerability to embolism is used as a proxy, have better drought adaptability. The other is the ability of embolism repair, that is, plants can restore the water transport capacity through embolism repair after recovering water conditions or when the evapotranspiration is relatively low at night [43]. The current understanding indicates that the recovery of xylem water transport function is mediated by the growth of xylem vessel, root pressure and osmotic regulation [17–19]. For short-term embolism repair, it is mainly achieved by the active repair mediated by root pressure and osmotic regulation. Root pressure is greater in the place closer to where root pressure is generated, therefore root pressure is more favorable for the repair of root embolism, which is faster than stem embolism repair. As for root system itself, in the dry season, the root pressure of deep roots is the greater than that of shallow roots, and most of them are positive. We found that the embolism recovery of deep roots was better than that of shallow roots. On the contrary, in the rainy season, the root pressure of shallow roots was greater than that of deep roots, which also led to the better embolism recovery of shallow roots than that of deep roots. This may be due to the higher temperature of the shallow soil. The establishment of root pressure requires the continuous supply of ATP by roots through respiration that may vary with soil temperature. Thus, root pressures are positively correlated with soil temperature [21].

The change of PLC in roots was greater than that in branches between midday and predawn, indicating that the root had stronger ability of embolism repair. At the same time, roots had higher xylem pressure than branches, consistent with the embolism repair ability, suggesting that the root pressure played an important role in the embolism repair. However, in the dry season, the xylem pressure of branches and even shallow root was negative over most of time, thus the embolism repair may not depend on the xylem pressure. In dry season, the low moisture content of shallow soil restricted the shallow root pressure to a great extent, and affected the short-term repair of shallow root embolism. In that situation, another mechanism of xylem embolism repair with active repair mediated by osmoregulation may play a role, which is usually closely related to the proportion of xylem parenchyma cells, soluble sugar concentration and other factors.

Our third question was whether the NSC is involved in the embolism repair. In this study, the soluble sugar content and the soluble sugar/starch ratio in deep roots were significantly lower than that in shallow roots and branches regardless in the dry season or the wet season. It is believed that the content of soluble carbohydrates in xylem, but not starch, is correlated with the capacity of embolism repair [29]. As stated above, in the dry season, the xylem pressure of branches, as well as shallow roots, may be not great enough to facilitate the embolism repair. Alternatively, the refilling of embolized vessels may have been associated with starch hydrolysis to soluble sugars in adjacent tissues [44]. At the same time, branches and shallow roots had high soluble sugar content and soluble sugar/starch ratio, which supports the mechanism of embolism repair with starch hydrolysis to soluble sugars. Thus, the mechanism of embolism repair may be different in different parts of plants [23]. Deep roots mainly depend on root pressure to refill the embolized vessels, while branches mainly depend on local osmotic pressure to do the work [44]. Shallow roots may use both of them to do the refilling. In the dry season, the embolism repair of shallow roots may more rely on osmoregulation with starch hydrolysis to soluble sugars, while in the wet season the repair may more depend on root pressure. The change of soluble sugar content in branches, shallow roots and deep roots may be related to the change of water potential in different parts. The soluble sugar content did not relate to the photosynthesis in different seasons (Supplemental Fig. 2), while the starch as well as NSC in wet season generally higher than that in the dry season, which may be related with photosynthesis. Starch content as well as NSC content in roots, especially in deep roots, were significantly higher than those in branches, even in the dry season, indicating that there was no carbon limit for maintaining plant water transportation and other physiological activities in the roots.

## Conclusions

In this study, the results indicate that deep root have greater water transport ability due to its larger vessel diameter, however more embolism vulnerability due to its bigger pit area proportion, larger individual pit area, and larger vessel diameter. There exists an obvious trade-off between water transport efficiency and safety for different organs to adapt the different environmental conditions. Although the root system, including the deep roots, has showed obvious embolism under the strong transpiration demand, the embolism of roots is not severe compared to the branches, which proved that the root system is not necessarily more prone to embolism in nature. This result also proves that xylem of a tree is a continuous system from roots to leaves, and vigorous transpiration can transmit water potential signal from crown to root tip. The relatively stable water condition in soil, especially in the deep layers, is favorable for the development of larger-diameter vessels in root xylem, however it can not prevent the root from forming embolism under the condition of high transpiration demand. The strong transpiration demand in summer, with the fluctuation of soil moisture, may limit the size of conduits in shallow roots and result in severe embolism. Nevertheless, roots are close to the place where the root pressure is generated, so the root has a strong ability to repair the embolism. The mechanism of embolism repair may be different in different parts of plants. Deep roots mainly depend on root pressure to refill the embolized vessels, while branches mainly depend on starch hydrolysis to soluble sugars to do the work. That the roots do not suffer the most severe embolism observed under natural conditions, is attributed to the relatively stable water condition in soil and frequent embolism repair through root pressure.

## Methods

### Plant material and field sites

This study was conducted in the Yellow River Xiaolangdi forest ecosystem research station (35° 01'N, 112° 28' E) located in Jiyuan, Henan province, China. The elevation is about 310 m, lying in the Taihang mountain foothill. The average annual temperature is 12.4–14.3℃, with the highest temperature in July or August, near to 30℃, the lowest in January, close to 0℃. The average rainfall is 641.7 mm, and varies seasonally under the influence of monsoon climate. Rainfall is concentrated in July ~ September, accounting for 68.3% of the annual precipitation. Seasonal drought usually occurs at the early growing season. Thus, the growing season of the area is divided into wet and dry conditions [23, 45]. This experiment was conducted in 2017, in which the precipitation was 205 mm from January 1^st^ to mid-July, and was 436 mm from mid-July to the end of December (Fig. 4).

This experiment was conducted in a 6-year-old walnut (*Juglans regia* L. cultivar Xiangling) plantation with 4 m between plants in the rows and 5 m between rows in north–south orientation. The walnut seedlings were purchased from Jiyuan nursery stock Co., Ltd. (Henan, China). The experimental plot is about 0.5 ht. In the middle of the experimental plot, three rows with a total of 60 plants were selected, of which three plants at both ends of each row were eliminated to avoid edge effect, and the remaining 40 walnut trees were used in this experiment. The walnut trees were 3–3.5 m in height, 7–9 cm in basal diameter, and 2.5–3.0 m in crown width. The soil at the site is about 1 m thick, below which un-weathered limestone bedrock exists. No irrigation was carried out on the experimental site. The measurements were taken in early July (dry season) and late August (wet season), respectively. In this study, the roots in different soil depths, as well as the branches, were used to compare their anatomy and hydraulic structure. With excavation we got access to deep root only about within one meter, for deeper it was hard to find any roots of this species for the 6-year-old trees. Thus, we referred to the roots in around 15 cm soil layer as shallow roots, and the roots in around 60 cm soil layer as deep roots.

### Meteorological data and soil moisture content

An automatic weather station was set in the walnut plantation to continuously detect air temperature and rainfall. The soil moisture content at the depths of 15 cm and 60 cm was measured by a portable time-3 TDR soil moisture tester (IMKO Ettlingen, Germany).

### Water potential, PLC and vulnerability curves measurements

A PMS pressure chamber (PMS 1505D-EXP, Albany, USA) was used to measure twig predawn water potential in the early morning before sunrise (5:00–5:30) and midday water potential in 12:00–14:00 at noon. Three twigs from each of tree were collected, immediately measured and values averaged. Three trees were measured. One-year-old branches in the middle-upper canopy on sunny side, and roots from shallow and deep soil layers were used to determine percentage loss of conductivity (PLC) and vulnerability curves. PLC measurement was carried out by using the pressure-flow method [35]. In predawn or at noon, approximately 40–50 cm long samples with the basal diameter of 0.6–0.8 cm of branches, shallow roots in soil depth of 10–20 cm, and deep root in soil depth of 50–70 cm were collected, by cutting under water. After the samples were collected, they were all wrapped with black plastic cloth. All the samples were submerged into tap water immediately and then taken back to the lab for PLC determination. The time from sampling to measurement should be controlled within 45 min. Our preliminary experiment showed that there was no any time impact on PLC measurements within the time range. PLC was measured by using three biological replicates with three segments averaged in each replicate. A 4-cm-long segment was cut underwater and used for PLC measurement. The segment was connected to a pressure-flow apparatus [35]. The balance was used to collect and weigh the solution to determine the flow rate through the segment. The native hydraulic conductivity (K_i_) was measured gravimetrically by determining the flow rate of the KCl solution at a pressure differential of 4 kPa. The stem segment was then flushed for 10 min in order to remove air embolisms at a pressure of 0.175 MPa. The hydraulic conductivity was then determined again at a pressure differential of 4 kPa and set as the maximum hydraulic conductivity (K_max_). The PLC was calculated as follows:

$$\mathrm{PLC}\:=\:100\:\times\;\left(K_{max}-K_i\right)/K_{max}$$

The vulnerability curves (VC) were measured by the air injection method [36, 37]. A segment of 30-cm-long branch and 40-cm-long root were cut underwater. The bark from the central portion of the segment was removed, and the segment was then mounted on the pressure collar (PMS 1505DEXP, Albany OR USA) and connected to the pressure-flow apparatus [46]. All the segments were flushed with 0.025 mol L^−1^ KCl solution for 20 min at 0.175 MPa pressure and the initial and also maximal conductivity (K_max_) was measured at a pressure differential of 4 kPa as described above. The segment was then pressurized by forcing compressed air into the chamber in a series of increasing pressure (increased by 0.5 MPa each time) so as to obtain a series of gradually increasing embolism. The test pressure was held for 15 min every time and then released to atmospheric pressure and equilibrated for 10–180 min underwater until gas was no longer released from the ends of the segment. The equilibrium time is mainly dependent on pressure, and thus increases with the increase of pressure applied. After equilibration, hydraulic conductivity (K_h_) was measured. The degree of embolism (percentage loss of conductivity, PLC) after pressurizing was calculated as (1 − K_h_/K_max_) × 100%. The air pressure applied to the chamber collar was successively increased for four to five times until PLC reached 80–100%. Vulnerability curves were plotted by percentage loss of hydraulic conductivity (PLC) *vs* injection pressure (P) with 3 trees. These curves were well-fit by the sigmoid equation [46, 47]:

$$\mathrm{PLC}\:=\:100/\left(1\:+\:\exp\left(\mathrm a\left(\mathrm P\:-\:\mathrm b\right)\right)\right)$$

Where a represents curve slope, and b indicates the point of the curve (along the x-axis) at which 50% of PLC occurs.

### Xylem pressure

Xylem pressure was measured at the same dates as water potential and PLC measurements with 3–4 trees according to the method of Améglio et al. [48]. A branch or a lateral root was cut off at a distance of 10 cm from its base, then, one cm of bark band was stripped off below the cutting of the remaining branch or root, and the bark edge was coated with silica gel to prevent desiccation. After the cutting was trimmed with a fresh razor blade, a hard-wall silicon tube (5 cm long) was connected to the cutting end and filled with degassed distilled water, which was left for overnight to equilibrate the xylem pressure. After then the other end of the silicon tube was connected to a pressure transducer (CX136-4, OMEGA, CT, USA). Continuous xylem pressures were recorded via a data logger (Cambell CR1000, USA) at 30-min intervals for a minimum period of 24 h.

### Nonstructural carbohydrates (NSC) determination

Branch and root samples for nonstructural carbon determination were collected from four trees respectively in early-July (dry season) and late August (wet season). The branch and root samples had the same diameters as those for PLC measurement. The samples were oven-dried at 70 °C for 2–3 days, and then ground and sieved through a 100-mesh screen. The sample powder (0.1 g) was poured into a 10 ml centrifuge tube, and 5 ml 80% aqueous ethanol (volume ratio) was added and incubated in a water bath at 80 °C for 30 min to extract soluble sugars (SS). The extracting solution was centrifuged at 3500 rpm for 10 min and the supernatant was transferred to a 100 ml volumetric flask. The precipitate was extracted twice again with 80% ethanol and the supernatants were pooled together. Total soluble sugars were determined photometrically at 620 nm on the supernatant in the presence of anthrone–sulfuric acid reagent [49]. The residue after extracting SS was hydrolyzed from starch to SS (glucose) with perchloric acid solution. Two milliliters of distilled water were added to the remaining residue, and the mixture was boilled in a water bath for 15 min. After cooling, 2 ml of 9.2 mol L^−1^ perchloric acid solution was added to the mixture and reacted for 15 min. The mixture was centrifuged at 3500 rpm for 10 min, and the precipitation was degraded with 2 ml of 4.6 mol L^−1^ perchloric acid for another 15 min and centrifuged as the above steps. The precipitate was rinsed 2–3 times with 5–6 ml distilled water. All the supernatants were pooled to a 100 ml volumetric flask. The soluble sugar concentration was determined as the above, and was referred to starch concentration. Soluble sugar (SS) and starch (St) concentrations (mg g^−1^) were calculated as content of the measured pool divided by dry weight of the sample.

### Microstructural determination

The size and sampled location of branches or roots used for vessel microstructural measurement were the same as those for PLC measurement. Samples were collected in August, 2017. A branch or root segment was sampled from a tree and a total of three trees were used, and the samples were naturally dried at room temperature.

Branch and root samples were cut into transverse sections, which were observed and photographed under a microscope to determine the vessels diameter and density [50]. For a transverse section, the current-year growth ring was selected and three visual fields were photographed and measured. More than 200 vessels per sample were measured. For vessel density measurement, six visual fields of each sample were photographed. After photographed, the vessel density was calculated.

The samples were also longitudinally cut into small blocks (5–10 mm in length) to expose radial surfaces. The blocks were air-dried and subsequently coated with gold in a sputter, and the samples were examined under a scanning electron microscopy (SEM) (Hitachi S-4800 Tokyo, Japan) for vessel inner wall ultrastructure. For each sample, 3–4 blocks were examined and photographed. The photos were used for the measurements of pit membrane area with a minimum of 150 pits and pit area ratio with six areas of each sample [51]. IMAGE J software was used for image analysis.

### Statistical analyses

All values were calculated as mean ± standard deviation (SD). One-way ANOVA was applied to test differences in parameters for dry and wet season. Sample sizes for all measurements were 3–5 replicates. Because it was difficult to obtain root samples, the replicates were limited. The significant difference was set at α = 0.05. The statistical analyses were all performed with SAS (SAS Institute Inc., Cary, NC, USA).

## Supplementary Information

**Additional file 1.** 

## Data Availability

All data generated or analyzed during this study are included in this article and available from the corresponding author on reasonable request.

## Abbreviations

PLC: Percentage loss of conductivity
P_50_: The water potential associated with 50% loss in hydraulic conductivity
NSC: Nonstructural carbohydrates
