# Supplementary material for: Xylem structure and hydraulic characteristics of deep roots, shallow roots and branches of walnut under seasonal drought
Source: BMC Plant Biol. 2022 Sep 14;22:440. doi: 10.1186/s12870-022-03815-2 (PMC9472371; doi:10.1186/s12870-022-03815-2)
Supplement: Supplementary file 1 — Additional file 1. [file 12870_2022_3815_MOESM1_ESM.docx]

Supplementary figure 1. Root hydraulic conductivity of shallow root and deep root in dry and wet season. Means ± standard deviations (n = 3) are shown, and different letters refer to significant difference in the same season at P < 0.05.

Radial root hydraulic conductivity was significantly greater in wet season than that in dry season regardless of the root depth (Suppl fig. 1). While the radial root hydraulic conductivity for deep root system was significantly greater than that of shallow root system in dry season, there was no significant difference in the radial root hydraulic conductivity between shallow roots and deep roots in wet season.

Supplementary figure 2. Photosynthetic rate, stomatal conductance, transpiration rate and water use efficiency of walnut in dry and wet season. Means ± standard deviations (n = 5) are shown, and different letters refer to significant difference at P < 0.05.

The photosynthetic rate, stomatal conductance, and transpiration rate of leaves in the dry season were significantly lower than those in the wet season, and the water use efficiency in the dry season was significantly higher than that in the wet season. The photosynthetic rate, stomatal conductance, and transpiration rate in the dry season were 62.5%, 59%, and 51% of those in the wet season, respectively.
